# DEAD-box helicase intrinsically disordered domains and structural dynamics of HIV-1 RNA are required to reveal DDX3X catalytic efficiency

**DOI:** 10.1093/nar/gkaf834

**Published:** 2025-08-28

**Authors:** Nathalie Chamond, Grégoire de Bisschop, Lisa Faria, Yamina Laoudi, Valerii Martynov, Bruno Sargueil

**Affiliations:** Université Paris Cité, CNRS, CiTCoM Unit, PARIS F-75006, France; Université Paris Cité, CNRS, CiTCoM Unit, PARIS F-75006, France; Université Paris Cité, CNRS, CiTCoM Unit, PARIS F-75006, France; Université Paris Cité, CNRS, CiTCoM Unit, PARIS F-75006, France; Université Paris Cité, CNRS, CiTCoM Unit, PARIS F-75006, France; Université Paris Cité, CNRS, CiTCoM Unit, PARIS F-75006, France

## Abstract

DEAD-Box helicases are enzymes that bind and remodel RNA and ribonucleoproteins. They are involved in almost every step of RNA metabolism. DEAD-Box helicases are thus major players of gene expression (dys)-regulation and intracellular parasite invasion such as retroviruses. Among many implications in pathologies, the human DEAD-Box helicase DDX3X is hijacked by HIV-1 at various steps including viral RNA export from the nucleus and translation initiation, but little is known about the way it interacts with the viral RNA as well as the structural consequences of this interaction. Here, we show that DDX3X binds to specific regions of HIV-1 5′UTR and dissociates tightly bound dimers of HIV-1 RNA. Such enzymatic activity resulting in the destabilization of a complex structure in multiple turn-over conditions has never been observed with a DEAD-box helicase. DDX3X-induced dynamics was followed using time-resolved structure probing, while footprinting revealed DDX3X preferential binding sites. By coupling the biochemical analysis of DDX3X enzymatic activity the systematic probing of HIV-1-derived RNAs structure, we challenge both the accepted structural model of HIV-1 genomic RNA dimers as well as the dogma considering DEAD box proteins as inefficient and rather promiscuous towards their RNA substrates. An explicative mechanistic model is proposed.

## Graphical Abstract

**Graphical Abstract:**
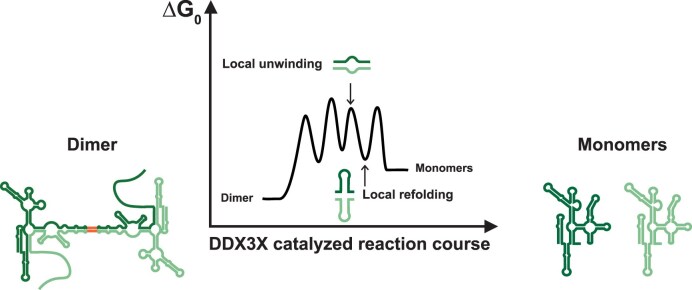

## Introduction

The DEAD-box proteins, which are the largest and most ubiquitous family of RNA helicases in all kingdoms of life, are implicated in all cellular processes involving RNA [1–3]. The DEAD-box proteins are ATP-dependent RNA binding proteins and RNA-dependent ATPases that have been shown to facilitate RNA folding as RNA chaperones, to remodel ribonucleoproteins, and to displace short RNA–RNA and RNA–DNA duplexes *in vitro*; but they are not processive, and they generally have shown little or no substrate specificity [2, 4]. The human DEAD-box protein DDX3X belongs to the Ded1/DDX3 subfamily whose members can act as general translation regulators which modify gene expression through remodeling particularly structured 5′UTRs in specific messenger RNAs (mRNAs) [5–7]. DDX3X is required for various viral infections (reviewed in [9]), and its mutations or dysregulation can result in various diseases including cancer (reviewed in [8]). Specifically, DDX3X is an essential host factor for HIV-1 replication, involved in several steps of the viral cycle through facilitating nuclear export of HIV-1 genomic RNA (gRNA) [10], the translation of viral proteins [11, 12], as well as through the formation of subcellular structures needed for HIV-1 replication [12]. Interestingly, the overexpression of or mutations in DDX3X lead to the spontaneous formation of stress granules (SGs) suggesting a role as SG assembly factor [13–15]. In light of its association with HIV-1 gRNA fate, DDX3X is currently considered as a valid target for the development of prophylactic and therapeutic antiviral drugs [16, 17].

**Figure 1.**
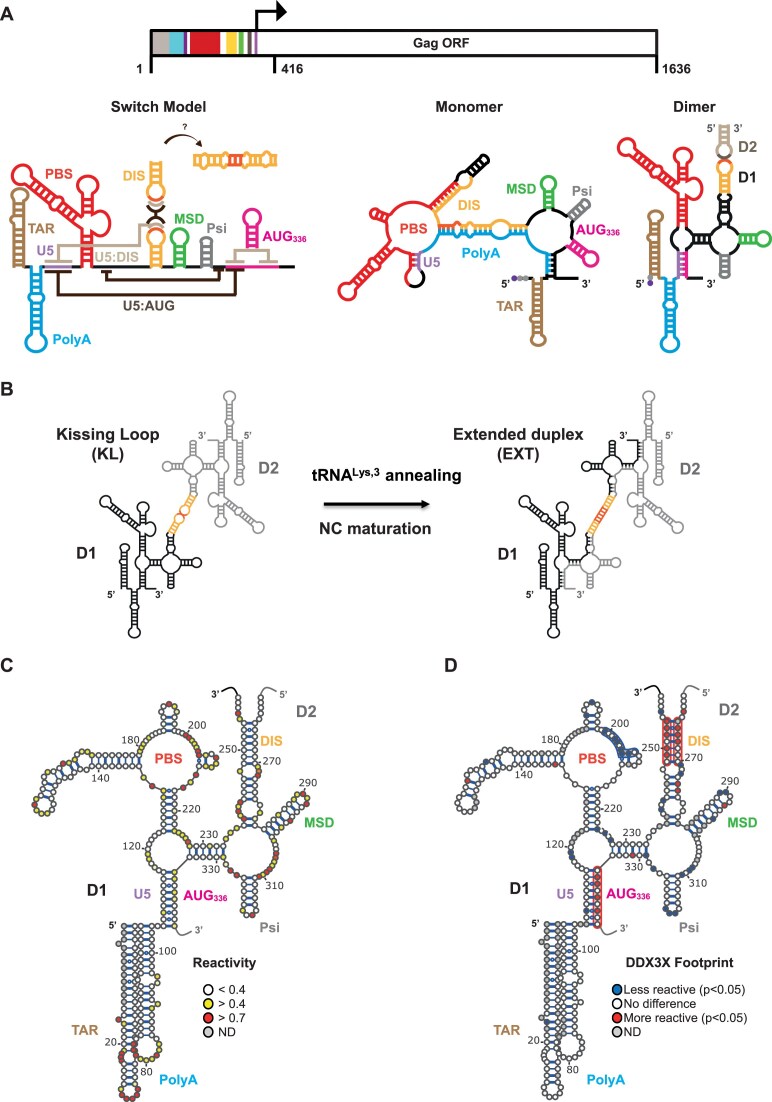
DDX3X binds to HIV-1 5′UTR and destabilizes the DIS stem-loop. (**A**) Schematic representations of the secondary structure models of HIV-1 5′UTR. The Switch Model [56–58] proposes that U5 interaction with either DIS (U5:DIS) or AUG (U5:AUG) favors the monomeric (light brown) or the dimeric conformation respectively (dark brown). Monomer and dimer indicate the secondary structure models as described in [34] explicating the influence of the TSS (guanosine at the 5′ end indicated by gray dots, cap in purple) on HIV-1 gRNA structure [32]. (**B**) During the viral replication and particle maturation, HIV-1 gRNA undergoes important structural rearrangements involving tRNA^Lys, 3^ annealing to the PBS and nucleocapsid (NC) maturation that allow the transition between poorly stable KL dimers [59] to give rise to the Extended (EXT) dimer conformation [41, 42]. (**C**, **D**) BzCN probing was performed by incubating 50 nM of RNA_1–1636_ in the absence (**C**) or presence (**D**) of 1 μM DDX3X_DQAD_ (or 1 μM MBP as a control). The secondary structure of HIV-1 5′UTR was modeled with IPANEMAP using the measured reactivities. The long-range pairings 105–115:335–345 (U5:AUG) and 228–233:329–334 corresponding to the dimer conformation were predicted. The DIS palindromic sequence is not fully represented in order not to prejudge the nature of the dimers (KL or EXT). (**D**) Differential reactivity map of DDX3X footprint. Reactivity differences in the presence and absence of DDX3X_DQAD_ are considered significant when the ratio is higher than 1.5 and the difference higher than 0.2 and the result of a *t*-test lower than 0.05, while the reactivity difference with MBP remains insignificant (*t*-test > 0.05). D1 and D2 refer to dimer strand 1 and 2, respectively. TAR (brown), Trans-Activation Response element; PolyA (cyan), PolyAdenylation signal; U5 (mauve), unique region; PBS (red), Primer Binding Site; DIS (orange), Dimerization Initiation Site; MSD (green), Major Splice Donor, Psi (gray), Core Packaging Signal; AUG_336_ (pink), Gag start codon.

The full-length HIV-1 RNA is a 9.2-kb capped and polyadenylated transcript containing a fairly long 5′UTR (335 nt) that harbors stable structures involved in the different steps of the viral cycle (Fig. 1A). In the host cell cytoplasm, the monomeric form of HIV-1 RNA serves as messenger RNA (mRNA) for the production of the Gag and Gag-pol polyproteins while the dimeric form of HIV-1 RNA serves as a gRNA to be encapsidated into nascent virions (reviewed in [18]). In terms of translation, structures in the 5′UTR such as the highly stable TAR stem loop strongly inhibits the translation machinery recruitment to the 5′ cap structure [19, 20]. Importantly, HIV-1 gRNA can initiate translation through alternate mechanisms that allow for the direct recruitment of the ribosome close to or at the initiation codon, resolving the problem of the cap accessibility. This mechanism relies on peculiar sequences and/or structures known as internal ribosome entry sites (IRESs). Two functional IRESs have been identified in HIV-1 gRNA, one in the 5′UTR under temporal regulation [21–23] and one within Gag-ORF suggested to regulate aRF usage and immunogenic peptides production [24–28]. Therefore, gRNA translation relies on at least three modes of translation initiation that are dependent on the presence of several structural elements present within the 5′UTR or embedded within Gag-ORF. Besides translation, HIV-1 gRNA serves as a genome to be encapsidated as dimers into nascent virions. The actual model for HIV-1 gRNA dimerization involves *cis-*acting RNA elements present within the 5′UTR. The dimerization initiation sequence (DIS), which contains a palindromic sequence able to form interactions with the same DIS sequence from another gRNA, permits the formation of a kissing loop (KL) motif [29–31]. DIS availability is therefore at the center of the dimerization process and Fig. 1A depicts the current models underlying HIV-1 gRNA dimerization. The “Switch model” proposes that the conformational changes observed are the result of an alternative pairing of the U5 region that will favor either the monomeric (U5:DIS) or the dimeric (U5:AUG) conformation. Alternatively, recent reports suggest that the selection of the transcription start site (TSS) for HIV-1 gRNA—resulting in the presence of 1, 2, or 3G at the 5′ end—affects the structure of the 5′UTR and the availability of DIS [32–34]. In this model, only the 1G RNA preferentially adopts a conformation sustaining DIS accessibility which, in turn, regulates the tendency of HIV-1 gRNA to form KL dimers [32, 35]. Moreover, *in vitro* and *in vivo* data [36–39] are consistent with a model in which HIV-1 RNA genomes are first selected for packaging as poorly stable dimers (KL), which subsequently form stable dimers (EXT) during viral particle maturation (Fig. 1B [40]). Importantly, these steps are accompanied by the annealing of transfer RNA (tRNA^Lys, 3^) [41]. From these experiments, it can be concluded that HIV-1 gRNA adopts multiple conformations whose nature and dynamics remain elusive.

Importantly, self-driven structural rearrangements are unlikely sufficient to promote the observed conformational switches of HIV-1 gRNA, raising the interesting possibility of a contributing role for auxiliary factors. Intriguingly, HIV-1 does not encode RNA helicases and thus must co-opt cellular factors to ensure its replication. Several RBP, whether viral or cellular, have been shown to directly interact with the 5′UTR of HIV-1 gRNA. In particular, the HIV-1 NC protein NC has been shown to promote the formation of extended dimers [41, 42] through its RNA chaperone activity. Importantly, amongst the different host RNA helicases that are associated with HIV-1 gRNA, only DDX3X and, to a lesser extent, RHA (DHX9), directly interact with the 5′UTR of HIV-1 gRNA and are resident of virions [43–45].

In this context, we previously identified HIV-1 RNA as a biological substrate for DDX3X and demonstrated that DDX3X ATPase activity relies on HIV-1 RNA structural determinants as well as on the presence of N- and C- terminal extensions of the enzyme. This raises the interesting possibility that the interaction of DDX3X with HIV-1 RNA is not only specific but could also be functional [46]. In this study, we make use of two SHAPE reagents differing by their half-lives to specifically interrogate the structure of HIV-1 RNA. First, we conducted a complete analysis of DDX3X interaction with a model RNA substrate of HIV-1 gRNA dimers (RNA_1–416_) and unraveled the extent of DDX3X helicase activity. Second, we used an original approach whereby DDX3X specific activity towards HIV-1 RNA fragments is coupled to systematic SHAPE probing of the RNAs’ structure to guide the identification of underlying structural determinants. Altogether, this work not only challenges our conception of a DEAD-box protein capacity towards a biological substrate RNA but also unexpectedly challenges our conception of the nature of mature HIV-1 dimers.

## Materials and methods

### Proteins and RNA production

MBP-DDX3X wild-type (DDX3X_WT_) and its mutant MBP-DDX3X_DQAD_ and MBP-DDX3X_132-607_ were essentially expressed and purified as previously described [46]. *In vitro* transcription was conducted as previously described [47] using pNL4-3 as a template, 5′-TAATACGACTCACTATAGGTCTCTCTGGTTAGACCAGATCT-3′ as a forward primer and reverse primers are indicated in Supplementary file.

### Dimer unwinding assays

50 nM of radiolabeled RNA were incubated at 37°C in Helicase buffer (40-mM Tris, pH 8, 50-mM NaCl, 0.5-mM MgCl_2_, 2-mM dithiothréitol (DTT), 0.01% NP-40) for 5 min in the presence of 2-mM ATP/Mg. DDX3X or DDX3X_132-607_ were then serially diluted and added to the samples that were incubated for additional 5 min. Ten-microliter aliquots were then mixed with 2.5-μl native loading dye (final concentration: glycerol 5%, xylene cyanol 0.02%, bromophenol blue: 0.02%) on ice and loaded on a pre-cooled 4% 19:1 acrylamide-bisacrylamide gel. Native electrophoresis was conducted at 100 V in THE buffer [34-mM Tris, 57-mM HEPES, 0.1-mM ethylenediaminetetraacetic acid (EDTA)] or THEM whenever in the presence 2.5-mM MgCl_2_ for 1 h. Gels were fixed in 30% ethanol and 10% acetic acid, dried, and exposed. A similar procedure was adopted for the kinetic analysis, except that 10 μl aliquots were removed at different timepoints after addition of DDX3X, and stored on ice in the native loading dye until gel loading. Images were processed with Multi Gauge V3.0 (Fujifilm). The fraction of dimer was calculated as the ratio (Idimer – IBG)/(Idimer + Imonomer – 2 × IBG) where Idimer, Imonomer, and IBG stand for the intensity of the dimer and monomer band and of the background, respectively. For the initial kinetics, data points were fitted to a one phase exponential decay curve Y = (Y0 − NS) × exp(−K × X) + NS, where X is the time, Y the fraction of dimer, and Y0 and NS are constants. Vi was obtained as the product of K by the concentration of RNA. The number of dissociated dimers was determined as [dimer]i × (Y0 − NS), where [dimer]i is the initial concentration of dimer, and Y0 and NS the initial and final ratio of dimer, respectively. [dimer]i is given by [total RNA]/2 × initial fraction of dimer.

### Kinetic parameters determination

To compare DDX3X activity towards different HIV-1 RNA fragments at increasing substrate concentrations, we slightly modified our experimental conditions. 120 pmol of RNA were heat denatured for 2 min at 90°C and then placed on ice for 2 min. The refolding was performed by adding ¼ Helicase 5× Buffer and incubated for 30 min at 37°C. RNAs were serially diluted (50–400 nM), complemented with 2-mM ATP/Mg and an excess of anti-DIS oligonucleotide (2 μM). Reactions were initiated by the addition of 10-nM DDX3X, incubated at 37°C. Aliquots were removed at different time-points and the reaction was stopped by the addition of Stop Buffer 5× (10% sodium dodecyl sulphate, 50% glycerol, 2% xylene cyanol, 0.5-M EDTA, 1% bromophenol blue). Samples were loaded on 1% agarose gel prepared in TBM (89-mM Tris Base, 89-mM boric acid, and 0.2-mM MgCl_2_) containing EtBr and fractionated at 100 V for 40 min at 4°C. Gels were analyzed using the Azure 400 visible fluorescent imager and the software Mutli Gauge (Fujifilm). The signal quantification allowed us to obtain the dimer/monomer ratio at each time-point and derive kinetic parameters. The apparent affinity for the RNA substrate and the apparent rate of dimer unwinding were calculated by measuring the variation of initial velocities of the unwinding reaction, as a function of RNA concentration. Data were analyzed according to the Michaelis–Menten equation: V = (k.E_0_.S^n^)/(Km + S^n^), where k is the catalytic rate, E_0_ is the input enzyme concentration, S is the dimeric RNA concentration, and n is the cooperativity index. Experimental data (3–6 independent experiments) and statistical analyses were performed using GraphPad Prism.

### Structure probing by SHAPE

SHAPE probing was essentially carried out as previously described [48, 49], specific details are described below.

#### Footprinting by SHAPE

3 pmoles of RNA were diluted in helicase buffer and incubated at 37°C. After 5 min, various concentrations of DDX3X_DQAD_ diluted in helicase buffer were added. The reaction volume was 120 μl. After additional 5 min, the RNA was probed with 40 mM BzCN (or dimethylsumfoxyde (DMSO) for the mock reaction) for a few seconds. Probed RNA was purified by salt precipitation by adding 10% (v/v) of 5-M ammonium acetate, 20 μg of glycogen, and 250% (v/v) of ethanol. Samples were incubated at −20°C for 1 h and centrifugated for 30 min at 15 000 rcf in cold. Pellets were washed with 200 μl of ethanol 70%, vacuum-dried and resuspended in 10 μl of H_2_O. Reverse transcription was performed with primers 223_R and 416_R and samples were analyzed following our established procedures [50].

#### Time-resolved probing by SHAPE

15 pmoles of RNA were diluted in 360 μl of helicase buffer and incubated at 37°C. After 5 min, a 60 μl aliquot was removed and probed with 40 mM BzCN (t = 0 min) while 5 nM of DDX3X_WT_ were added to the remaining solution. Sixty-microliter aliquots were removed at t = 2, 5, 10, and 30 min and probed with 40-mM BzCN for a few seconds then placed on ice. Neat DMSO was used for control reaction. Probed RNA was purified and analyzed as previously described. Nucleotides were clustered according to their reactivity change using scikit-learn’s algorithm. Reactivites were first normalized to unit form. Nucleotides with an undetermined reactivity were excluded before the clustering. Nucleotides whose maximum normalized reactivity change (Rmax − Rmin)/(Rmax + Rmin) was <0.2 were considered as not sufficiently significant and were removed from the clusters. An additional absolute reactivity threshold of 0.2 was applied. Reactivities were averaged within each cluster at each timepoint along with the standard error of the mean. The code for nucleotide clustering is available at https://github.com/gdebissc/DDX3.

#### HIV-1 RNA fragments probing by SHAPE-CE

RNAs were probed with 1M7 following our established procedures [51], reverse transcribed with D4/D2 labeled primers (listed in Supplementary file) to cover the entire sequence of the RNA of interest. Mean reactivities and standard error of the mean were calculated from a minimum of three independent experiments at each nucleotide position. Data were analyzed using our in-house workflow, IPANEMAP Suite [52].

#### Base-pair probability prediction and fragment classification

RNAfold was used to derive base-pair probabilities from SHAPE data for each fragment with the following command: RNAfold -p –noLP –noPS –shape=“$SHAPE_FILE” < “$FASTA_FILE”. Output postscript files were parsed to extract base-pair probabilities. An extra-tree classifier was trained to predict fragment group (1 or 2) based on the base-pairs probabilities of the first 343 nucleotides. Because RNAfold only reports base-pairs with probability >10^−4^, missing base-pairs were assigned a probability of 0. The model was implemented using scikit-learn with default parameters. The features importance (Gini importances) were then extracted from the trained model. The scripts used in this analysis are available at https://github.com/gdebissc/DDX3.

## Results

### DDX3X specifically interacts with HIV-1 RNA

In order to examine the molecular details of DDX3X interaction with HIV-1 RNA, we first sought to map the binding sites of DDX3X on HIV-1 Gag RNA secondary structure (RNA_1–1636_). The DDX3X_DQAD_ mutant, a previously characterized mutant that lacks ATPase activity but retains its ability to bind RNA, was used to conduct footprinting experiments [50]. This technique allows not only to detect binding sites, but also to observe potential structural rearrangements upon binding. In this study, HIV-1-derived RNAs were produced to start with 2G which correspond to the capped HIV-1 RNA + 1 TSS that displays an increased propensity to form dimers *in vivo* and *in vitro* [32, 34]. As can be observed in Fig. 1 and Supplementary Table S1), the reactivity profile obtained with BzCN in the absence of MBP-DDX3X_DQAD_ (further referred to as DDX3X_DQAD_) is consistent with most of the RNA being under a dimeric conformation. Several stretches of nucleotides distributed along the structure show a significant reactivity drop in the presence of DDX3X_DQAD_. An important cluster of footprinted nucleotides is observed within the PBS loop, indicating that DDX3X_DQAD_ specifically alters C_199_-C_206_ reactivity to the SHAPE reagent_._ Interestingly, the results also show some nucleotides whose reactivity increases in the presence of DDX3X_DQAD_, suggesting some structural rearrangement of HIV-1 5′UTR upon DDX3X_DQAD_ binding. These nucleotides are located within the DIS upper stem and around the AUG start codon (U_249_-G_254_; G_265_-C_267_ and A_336_-U_341_ in Fig. 1D). This observation suggests that those nucleotides are involved in interactions that can be destabilized upon binding of the helicase prior to ATP hydrolysis. This is consistent with a mechanistic model in which DEAD box helicases alternate between an “open” (unbound) and a “closed” (bound) conformation whereby the energy of ATP hydrolysis restores the “open” state resulting in the release of the substrate RNA [53–55]. Since the DIS stem loop and the nucleotides surrounding the AUG codon are involved in the monomer-to-dimer transition, and because in the experimental conditions the RNA is mostly dimer, we hypothesize that this may reflect the first events of dimer unwinding.

### DDX3X efficiently promotes HIV-1 RNA dimer dissociation

**Figure 2.**
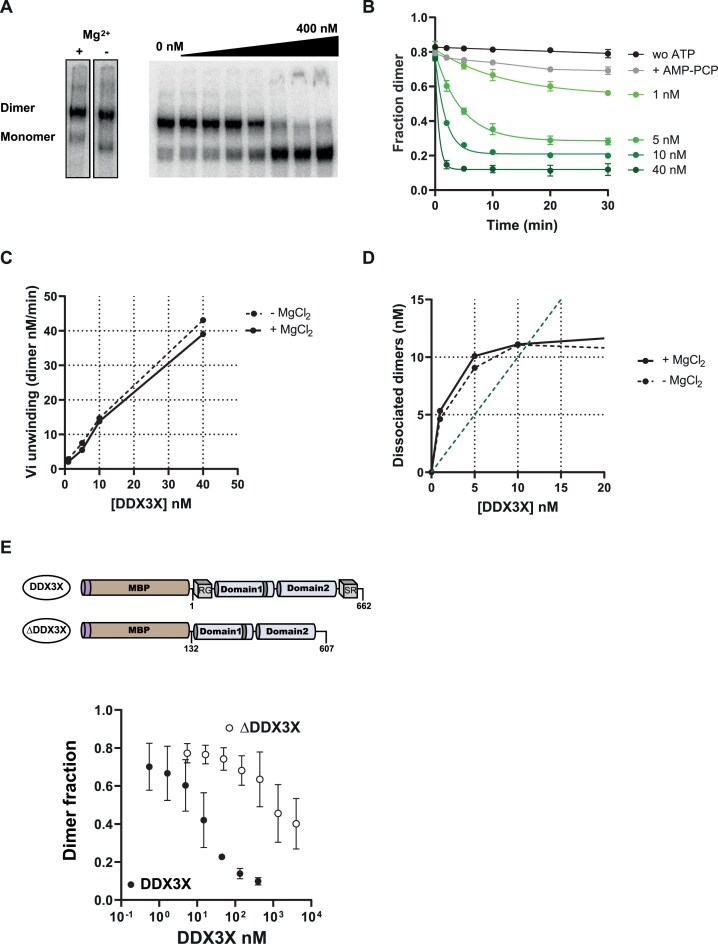
DDX3X actively dissociates HIV-1 5′UTR dimers in multiple turn-over. (**A**) HIV-1 5′UTR (RNA_1–416_) forms dimers *in vitro* that are stable in the absence of magnesium during separation. Fifty nanomolars of HIV-1 5′UTR were incubated for 10 min in the absence or in the presence of different concentrations of DDX3X_WT_ (0.5–400 nM) and separated by native gel electrophoresis. (**B**) Time-course of dimer destabilization upon incubation with increasing concentrations of DDX3X_WT_ (1–40 nM) in the absence (black line) or in the presence of 2-mM ATP (green lines) or of AMP-PCP (gray line). The fraction of dimeric RNA was assessed by native gel electrophoresis. (**C**, **D**) Analysis of the initial speed of the reaction and the total dissociated dimers. Dashed lines indicate the amount of dimer measured in the absence of Mg in the running buffer. Green dashed line represents [dissociated dimers] = [DDX3_WT_]. (**E**) Schematic representation of the DDX3X versions used. Different concentrations of DDX3X (DDX3X_1–662_; 0–400 nM) or of ΔDDX3X (DDX3X_132–607_, 0–4 μM) were incubated in the presence of 50 nM of RNA_1-416_ in the presence of ATP for 10 min and the RNA species were separated by native gel electrophoresis and quantified. Error-bars refer to the standard error of the mean of three vindependent replicates.

To assess the impact of DDX3X_WT_ on the stability of HIV-1 RNA dimers, we chose the model RNA_1–416_ and measured the dimer-to-monomer ratio in the presence of various amounts of the protein using native gel electrophoresis (Fig. 2 and Supplementary Table S2). As suggested by the footprint results, but to our surprise, DDX3X_WT_ efficiently dissociate HIV-1 RNA dimers. However, in the absence of ATP or in the presence of a nonhydrolysable ATP analog (AMP-PCP), DDX3X_WT_ fails to destabilize the dimers, confirming that ATP hydrolysis is required. Further, the effect of DDX3X_WT_ is even seen at a concentration lower than the RNA concentration, which is indicative that DDX3X_WT_ may achieve multiple turnovers (Supplementary Fig. S1). By deriving the number of dissociated dimers for various [DDX3X_WT_] inferior to [dimer] (see the ‘Materials and methods’ section) we confirmed that DDX3X functions in multiple turn-over, which to the best of our knowledge has never been observed with a DEAD-box helicase. Indeed, previous studies performed with short duplexes failed to highlight multiple turnover reactions [55], indicating that such model substrates do not reveal the full DDX3X activity. Considering the observed efficacy of DDX3X towards HIV-1 RNA dimers, we wondered whether the enzyme only destabilizes “KL” dimers (Fig. 1B). Such dimers can be experimentally distinguished from extended dimers because they require MgCl_2_ to be stable on native gels [60–63]. In order to test this hypothesis, dimers destabilized by DDX3X_WT_ were separated by native gel electrophoresis either in the presence of magnesium or in its absence. The observed initial rate as well as the number of dissociated dimers do not differ significantly when analyzed in absence or presence of magnesium, indicating that DDX3X is comparably active towards “stable” dimers (Fig. 2C and D). This result suggests that DDX3X activity induces the destabilization of an extended duplex involving at least 63 intermolecular base pairs which represent a ΔG^0^ of ∼−70 kcal/mol (EXT; Fig. 1B).

The N-and C-terminal extensions of DDX3X are intrinsically disordered regions (IDR) involved in liquid–liquid phase separation and SG formation [14, 64–66]. These terminal extensions are required for DDX3X specific binding as well as its specific ATPase activity [46]. Therefore, we investigated the role of the helicase terminal extensions by assessing the activity of the truncated DDX3X_132–607_ (ΔDDX3X) on RNA_1–416_ dimers. As shown in Fig. 2E, the removal of the terminal extensions led to a 100-fold decrease in DDX3X activity. This latter observation aligns with previous results showing that, in contrast to the full-length protein [46], this truncated version of DDX3X presents a poor affinity for RNA, an absence of specific stimulation of its ATPase activity, as well as a reduced activity on short duplexes [67]. These findings emphasize the key role of DDX3X’s terminal extensions for its specific enzymatic functions.

### Analysis of the 5′-UTR remodeling upon DDX3X interaction

**Figure 3.**
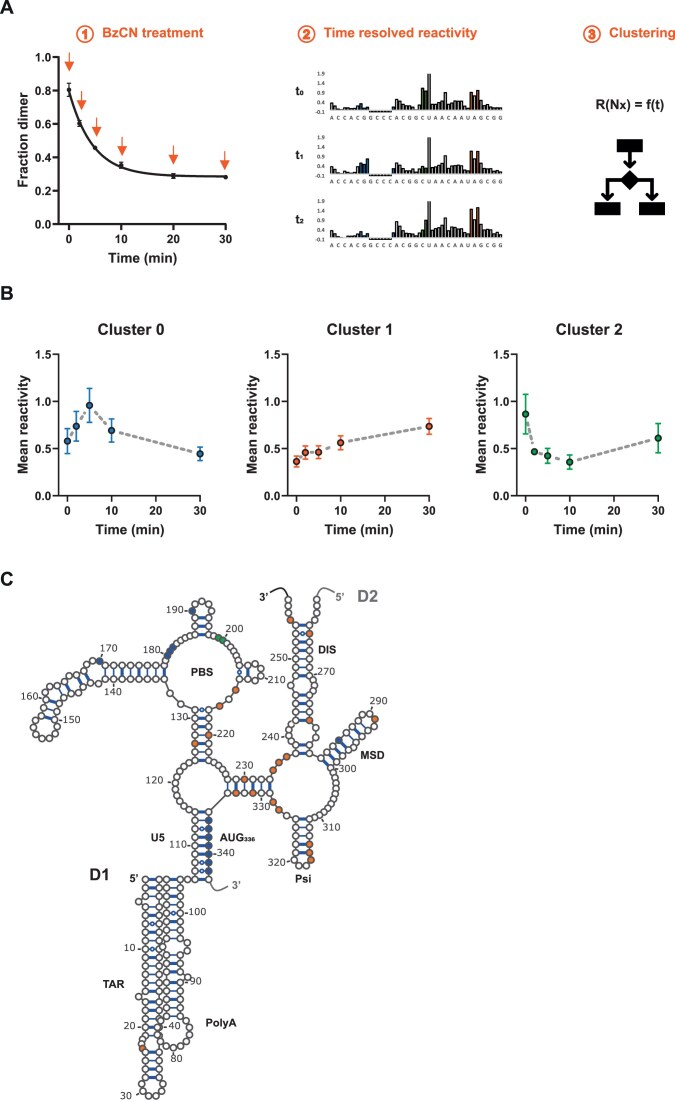
DDX3X activity remodels the structure of HIV-1 5′UTR. (**A**) Schematic of the approach used to identify structure changes induced by DDX3X activity. Left panel: 50 nM of HIV-1 5′UTR (RNA_1–416_) were incubated in the presence of 5-nM DDX3X and the dimer-to-monomer transition was probed with BzCN at different timepoints. Middle panel: Probed RNA was purified and reverse transcribed to measure nucleotides reactivity. Right panel: Nucleotides were clustered according to their reactivity change. (**B**) Average nucleotide reactivity at each timepoint within each cluster. Error bars refer to the standard error of the mean. (**C**) Secondary structure of HIV-1 5′UTR dimer showing the position of the nucleotides from the three clusters. Nucleotide color indicates cluster number as in panel (B). D1 and D2 refer to dimer strand 1 and 2, respectively.

These findings prompted us to investigate the structural rearrangement leading to the dissociation of dimers. We designed a set of time-resolved probing experiments to follow HIV-1 5′UTR conformational switch induced by DDX3X. More precisely, we probed RNA_1–416_ at different time points during the incubation with DDX3X_WT_ with the fast reacting SHAPE molecule BzCN (Fig. 3). Because we wanted to observe the RNA conformational changes and not the footprint of DDX3X, the experimental conditions were set from the native gel analysis in order to maximize the number of dissociated dimers while minimizing [DDX3X_WT_]/[RNA] (DDX3X_WT_ 5 nM/RNA_1–416_ 50 nM). Consequently, the evolution of the measured reactivities reflect the average conformational rearrangement in the samples (Supplementary Fig. S2). The reactivity of most nucleotides does not significantly change with time. This agrees with previous studies indicating that some elements such as TAR, PolyA, and the PBS-TLE (tRNA Like Element) are present both in the monomer and in the dimer conformations. The remaining nucleotides were clustered according to the evolution of their reactivity with time (see the ‘Materials and methods’ section). This revealed that the nucleotides are differentially affected by DDX3X and could be classified into three clusters: those with increasing reactivity, either transient (cluster 0) or reaching a plateau (cluster 1), and those with a transient decrease in reactivity (cluster 2). Cluster 1 could reflect the conformational switch between the monomer and the dimer. As such, the increased reactivity around residues U_228_-C_233_:G_329_-A_334_ and of U_291_, is consistent with an in-gel probing experiment performed on monomer and dimers of a similar fragment (RNA_1–412_ [68]. For instance, it appears that there are many nucleotides exposed around Psi which suggests that this region undergoes an important conformational change. While significant reactivity changes within the DIS element have never been observed between monomer and dimer conformations, here we observe selective increases in reactivity for A_255_, G_265_, and G_277_ that could reflect the greater flexibility of SL1 (DIS) in the monomer conformation. In addition, cluster 0 or cluster 2 suggest the existence of conformational intermediate(s). An intriguing observation came from the nucleotides surrounding the AUG start codon. Their reactivity exhibits an increase within the first 5 min followed by a decrease down to the initial value (cluster 0). This suggests that DDX3X_WT_ triggers the unwinding of the AUG:U5 pairing so that the AUG nucleotides become accessible to the SHAPE reagent during the transition. In contrast, there is no significant change in reactivity for the nucleotides within the U5 element suggesting that they are immediately involved in another pairing right after AUG:U5 pairing destabilization. Conversely, nucleotides such as C_199_ et U_200_ within the PBS show a transient decrease in reactivity (cluster 2). This cannot result from DDX3X interaction as observed in the footprint experiments because of the protein/RNA ratio (1:10 DDX3X:RNA), but should rather reflect a structural rearrangement induced by DDX3X activity. This is consistent with the observation that 11 nucleotides within the PBS see their reactivity modified over the time course. Our observation could be coherent with a recent report identifying an alternative monomer conformation [34] that could be transiently attained by RNA_1–416_. The overall observed reactivity changes at multiple sites are consistent with an extensive remodeling of the 5′UTR by DDX3X and are reminiscent of the monomer-to-dimer switch model (Fig. 1A) whereby U5 interacts with either DIS (monomer) or AUG (dimer). Clearly our observation cannot be attributed to the simple destabilization of unstable KL dimers but rather reflects an EXT dimer to monomer transition.

### Determinants within Gag ORF hinder dimer destabilization by DDX3X

**Figure 4.**
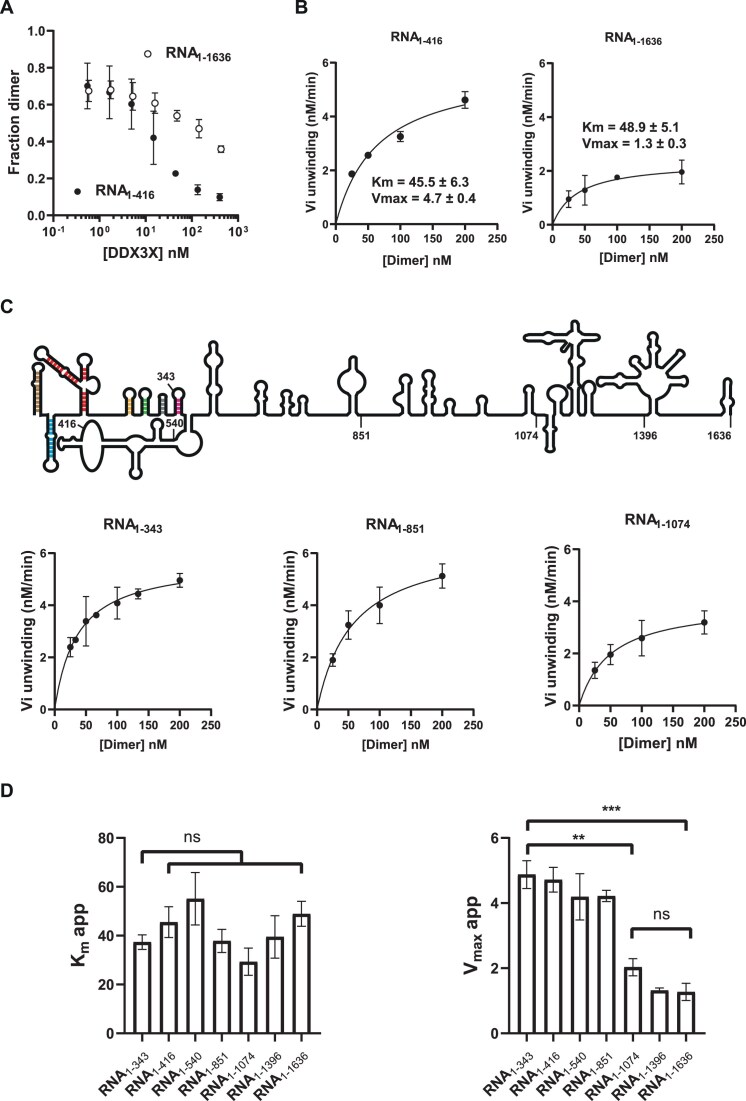
The presence of Gag-ORF reduces dimer destabilization by DDX3X. (**A**) Different concentrations of DDX3X_WT_ were incubated in the presence of 50 nM of RNA_1–416_ or RNA_1–1636_ in the presence of ATP for 10 min and the RNA species were separated by native gel electrophoresis and quantified. (**B**–**D**) Kinetic parameters determination of DDX3X activity towards 3′-deletions of RNA_1–1636_. Reactions were performed in the presence of 10 nM DDX3X, 50–400 nM RNA and 2-mM ATP in helicase buffer. The linear regression of monomer release versus time allows for the initial rate (**V**) determination at different RNA concentrations. DDX3X exhibits Michaelis–Menten kinetics with an apparent Km and Vmax that were determined for each RNA. The values represent the average and standard deviation of at least three independent experiments (see Supplementary Table S3). *t*-test values are indicated: nonsignificant, n.s; *P<.05; **P<.01, and ***P<.001. (**C**) Schematic representation of RNA_1–1636_ [69, 70] and 3′ truncations. The conserved elements of the 5′UTR are indicated. TAR, brown, PolyA, cyan, PBS, red, DIS, yellow, MSD, green, Psi, gray, and AUG, pink.

Interestingly, when assaying the effect of DDX3X_WT_ on a larger fragment, we discovered that dimers obtained with RNA_1–1636_ are much more resistant to DDX3X destabilization (Fig. 4A and Supplementary Table S2). This observation could reflect DDX3X titration by a “longer” RNA, an increased reassociation of the dimers after unwinding in the presence of Gag-ORF, or else that the presence of Gag-ORF increases dimers stability. In order to better dissect the differences between these two RNAs, we slightly modified our experimental conditions. To prevent reassociation of the dimers after DDX3X destabilization, experiments were performed in the presence of an excess of DNA oligonucleotide targeting DIS in the reaction buffer (Supplementary Fig. S3). In addition, experiments were carried out with saturating substrate concentrations. Then, we measured the kinetic parameters of DDX3X unwinding activity in the presence of each RNA (Fig. 4B and Supplementary Table S2). The results indicate that DDX3X unwinding activity follows Michaelis–Menten kinetics which is coherent with its previously described ATPase activity in the presence of HIV-1-RNA [46]. First, in the presence of the model RNA_1–416_, DDX3X displays an apparent k_cat, app_ of 0.9 min^−1^ confirming that the protein dissociates HIV-1 RNA dimers in multiple turn-over conditions. As can be observed in the figure, RNA_1–416_ and RNA_1–1636_ display similar functional affinities (apparent K_m_ = 45.5 ± 6.3 nM and 48.9 ± 5.1 nM, respectively) suggesting that DDX3X is not titrated by nonspecific sites in the longer RNA. In contrast, the apparent V_max_ of DDX3X_WT_ measured in the presence of RNA_1–1636_ displays a nearly 4-fold reduction as compared to that of RNA_1–416_ (apparent V_max_ = 4.7 ± 0.4 nM min^−1^ and 1.3 ± 0.3 nM min^−1^ for RNA_1–416_ and RNA_1–1636_, respectively). These results suggest that the observed reduced unwinding efficiency of DDX3X towards RNA_1–1636_ is the consequence of structural elements embedded within Gag-ORF. In order to identify such element(s), we determined DDX3X_WT_ kinetic parameters towards 3′-truncated versions of RNA_1–1636_, i.e. RNA_1–1396_, RNA_1–1074_, RNA_1–851_, RNA_1–540_, and RNA_1–343_.

As reported in Supplementary Table S3, DDX3X displays a similar Km for the seven RNA tested, confirming that DDX3X functional affinity is equally supported by determinants embedded within HIV-1 5′-UTR (G_1_-C_343_). Interestingly, the apparent V_max_ distinguishes two groups of RNAs significantly differing by their susceptibility to DDX3X_WT_ destabilization (Fig. 4D, right panel), i.e. RNA_1–343_, RNA_1–416_, RNA_1–540_, and RNA_1–851_ as compared to RNA_1–1074_, RNA_1–1396_, and RNA_1–1636_. Altogether, these results indicate that DDX3X unwinding activity depends on the structure of HIV-1 RNA dimers and suggests the presence of a dimer-influencing element between U_851_ and A_1074_.

### Identification of a structural determinant influencing HIV-1 dimer structure

**Figure 5.**
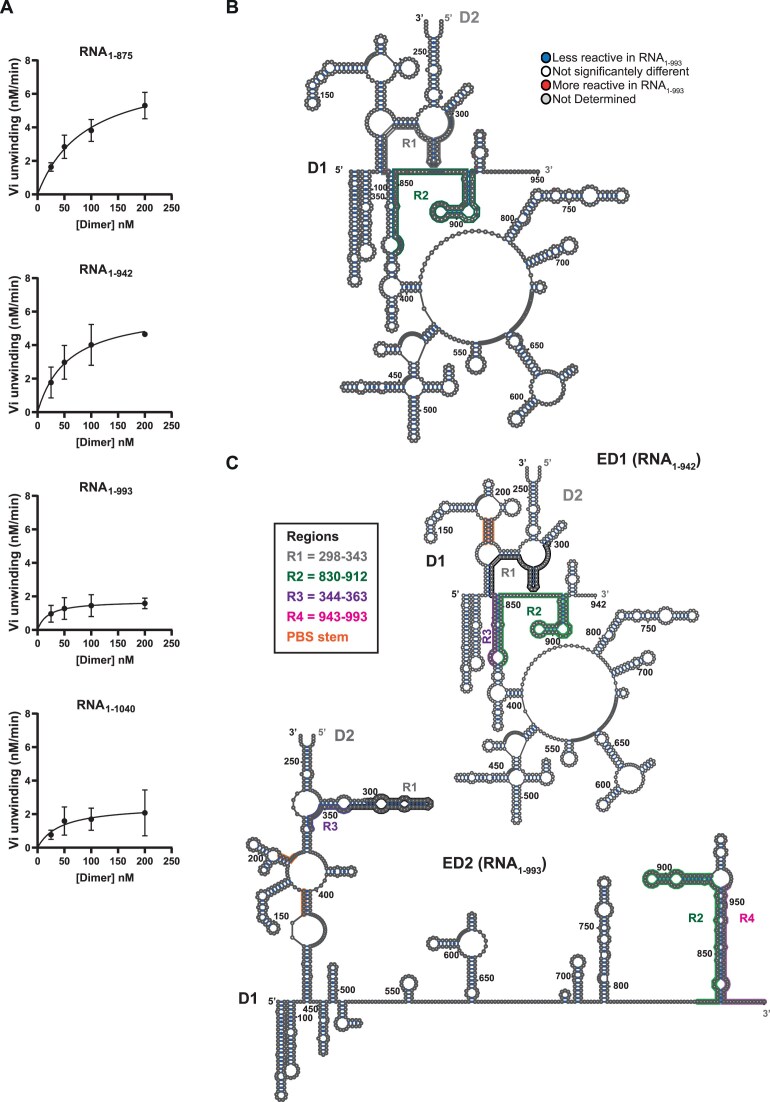
DDX3X unwinding rate identifies an alternative structural model for HIV-1 dimer. (**A**) Kinetic parameters determination of DDX3X activity towards RNA_1–875_, RNA_1–942_, RNA_1–993_, and RNA_1–1040_ (see Supplementary Table S2). (**B**) The comparative probing of RNA_1–942_ and RNA_1–993_ is reported on RNA_1-942_ structure as modeled by IPANEMAP. The two regions concealing significant differences are indicated: R1 (gray) comprising nt 298–343 and R2 (green) comprising nt 830–912.

In order to better identify the role of the 851–1074 region on DDX3X activity, we designed four new RNAs (RNA_1–875_, RNA_1–942_, RNA_1–993_, and RNA_1–1040_) and determined DDX3X apparent kinetic parameters in their presence (Fig. 5A and Supplementary Table S4). Although K_m_ values fluctuate according to the construction, they do not define statistically different groups and we suggest that such fluctuation reflects some conformational heterogeneity of some RNAs. In contrast, the apparent Vmax for these RNAs clearly discriminates two groups, RNA_1–875_ and RNA_1–942_ on one hand and RNA_1–993_ and RNA_1–1040_ on the other hand. These results allow to narrow down the identification of an RNA determinant critical to DDX3X destabilization of HIV-1 RNA dimers to a 50 nt window (942–993). We therefore probed the structure of both RNA_1–942_ and RNA_1–993_ by SHAPE and secondary structure models were issued by IPANEMAP (Supplementary Fig. S4). As expected, most of the nucleotides present similar reactivity values with the exception of two specific regions R1 (spanning A_298_–A_343_) and R2 (spanning C_830_-G_912_), the reactivity of which is essentially decreased in RNA_1-993_ suggesting an important structural rearrangement of this region in the presence of nt 943–993 (Fig. 5B). As depicted in Fig. 5C, two models emerged from this analysis. In model ED1 (corresponding to RNA_1–942_), while the 5′UTR corresponds to the dimer conformation depicted in Fig. 1A, Gag-ORF is modeled as an important structure concealing G_344_-C_853_ in the stem involving G_344_-C_403_ (in purple) and G_821_-C_853_ (in green). Concerning Model ED2 (corresponding to RNA_1–993_), the 5′UTR contains conserved motifs such as TAR, PolyA, the PBS/TLE, and DIS but also new structures. Notably, this model proposes an interaction between the MSD and the AUG region (highlighted in gray) which agrees with the changes observed in the reactivity profiles (R1). In addition, the PAS (Primer Activation Signal) stem is proposed to interact with G_401_-G_407_. Strikingly, U_107_-U_122_, containing the U5 element, forms a strong helix with A_431_–A_446_. Moreover, C_946_–C_981_ (in pink) sequesters part of the G_821_-C_853_ stem (R2, in green) to form an alternative G_837_-C_981_ structure. Taken together, these models are coherent with an indirect role of nt 942–993 whose presence would allow the inclusion of nt 336–446 in the intermolecular association and a remodeling of the 5′UTR unfavorable to DDX3X destabilization (see the ‘Discussion’ section).

### DDX3X helicase activity reflects HIV-1 dimer structure

**Figure 6.**
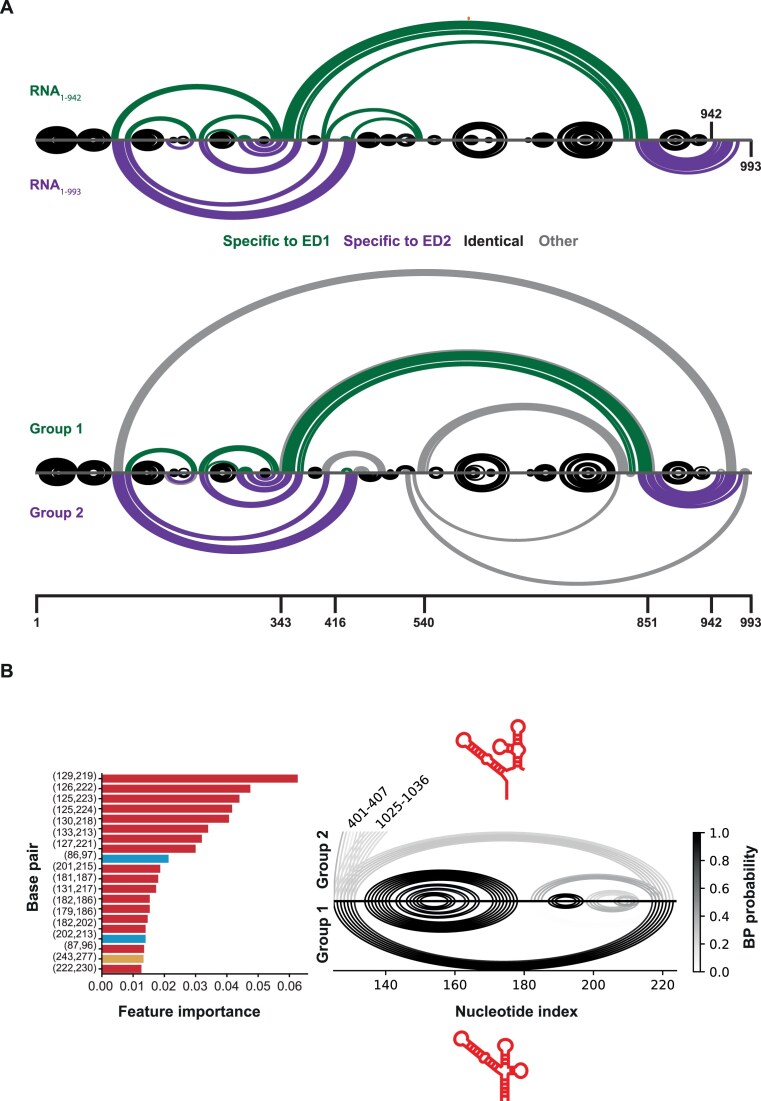
HIV-1 RNA structure discriminates dimer susceptibility to DDX3X destabilization. (**A**) Comparison of the structural models obtained for RNA_1–942_ and RNA_1–993_ as compared to models obtained for RNAs that are more sensitive (group 1) or more resistant (group 2) to DDX3X destabilization. (**B**) Left panel: Features importance from the Extra-Trees classifier for the 20 most discriminative 5′UTR base-pairs distinguishing group 1 and group 2. Colors indicate the secondary structure elements as in Fig. 1: PBS (red), PolyA (cyan), and DIS (yellow). Right Panel: Median base-pair probabilities across fragments of group 1 (bottom) and group 2 (top) for the nucleotides within the PBS.

To evaluate whether the structural difference could apply to the different RNAs tested in this study, we performed the probing of RNA_1–343_; RNA_1–416_, RNA_1–540_, RNA_1–851_, RNA_1–1074_, RNA_1–1396_, and RNA_1–1636_ in the same conditions (Supplementary Table S1). We took advantage of IPANEMAP, our recently developed modeling pipeline that can consider multiple datasets to model one RNA [52, 71]. We modeled RNA_1–993_ structure using probing data for nucleotides comprised within 1–993 for RNAs that are sensitive to DDX3X destabilization (group 1, comprising RNA_1–343_, RNA_1–416_, RNA_1–540_, RNA_1–851_, and RNA_1–942_) on the one hand, or from RNAs that are more resistant to DDX3X destabilization (group 2, comprising RNA_1–993_, RNA_1–1074_, RNA_1–1396_, and RNA_1–1636_) on the other hand. As can be observed in Fig. 6A the models obtained for each group parallel those obtained for RNA_1–942_ and RNA_1–993_ (ED1 or ED2 conserved interactions are depicted in green or purple, respectively). This result shows that ED1 and ED2 models are representative models of group 1 and group 2 RNAs and confirms that the extent of DDX3X rate of unwinding correlates with the structure of the dimer. We next search for a structural signature within the 5′UTR discriminating between group 1 and group 2 RNAs. We used RNAfold, guided by our SHAPE data, to generate probabilities for base-pairs within the 5′UTR for each RNA. We looked for those that discriminate against both groups. To this end, we trained an Extra-Trees classifier using the base-pair probabilities as input features to predict group belonging (1 or 2). Interestingly, this analysis revealed the PAS base-pair probabilities as the most discriminative features for the two groups (Fig. 6B).

## Discussion

### DDX3X efficiently destabilizes extensive base pairing holding HIV dimers

In this study, we demonstrate that DDX3X performs multiple turn-over reactions (at least 5–10 under our reaction conditions) which is an unprecedented observation, not only for DDX3X, but for DEAD-box proteins in general. In conditions of saturating concentrations of ATP and RNA substrates, the apparent k_cat, app_ is 0.9 dimer min^−1^. As ATP hydrolysis rate in presence of RNA is K_cat, app_ ≈ 20 min^−1^ [46], this suggests that RNA dimer destabilization requires about 18 cycles of ATP hydrolysis equivalent to 131.4 kcal/mol (the free energy of ATP hydrolysis in aqueous solution under standard condition is 7.3 kcal/mol [72]. In group 1 and group 2 models, the dimers are zipped by 63 and 91 base pairs, respectively, corresponding to −89.7 kcal/mol and −111.8 kcal/mol as calculated by RNAEval [73] (Supplementary Fig. S6). Therefore, the energy spent to unfold one dimer appears consistent with the destabilization of the extensive pairing proposed to be involved in the dimer formation. The destabilization of such large duplex structures is not incompatible with the prevailing mechanistic model of DDX3X, which characterizes it as a distributive process limited to short RNA duplexes, but challenges it in some aspects.

**Figure 7.**
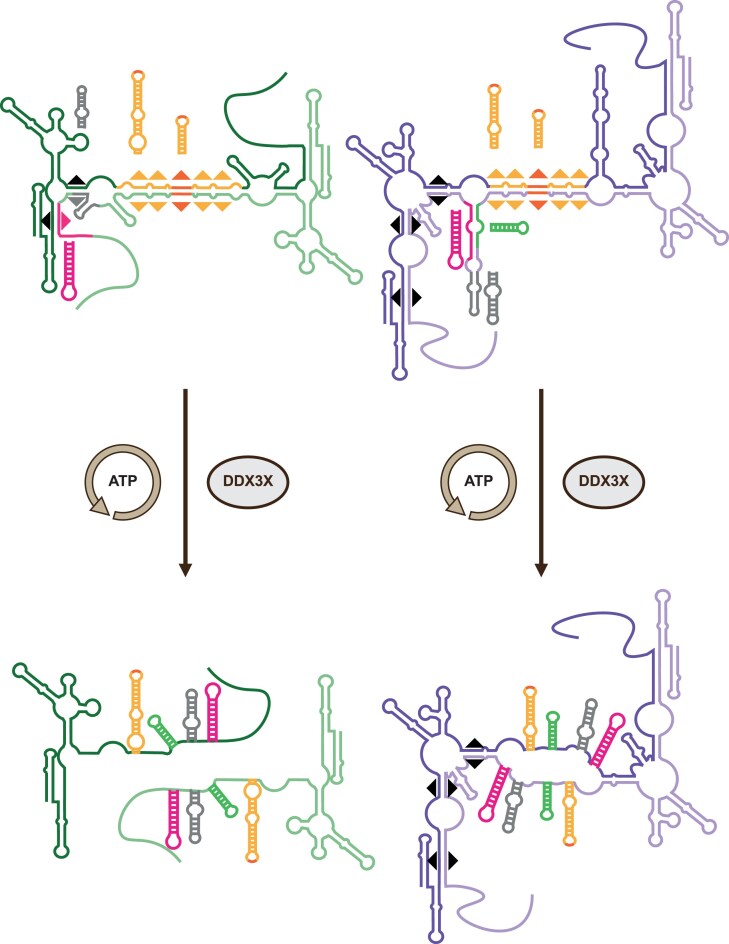
An explicative model for the differential activity of DDX3X towards HIV-1-derived RNAs. The structure of group 1 RNAs (in green) favors monomer stabilization through local structures formation upon DDX3X action while group 2 RNAs (in purple) local structures are not sufficient for monomer stabilization. In group 1, the intermolecular interactions involving DIS (SL1, yellow), Psi (SL3 extended, gray), and AUG (SL4, pink) can refold into local intramolecular structures while in group 2 this property is restricted to DIS (SL1, yellow). MSD (SL2, green) is also indicated. Black arrows indicate regions that could reduce DDX3X apparent rate of unwinding.

We propose a multi-step model to explain this observation (Fig. 7), wherein DDX3X initiates unwinding at localized, short intermolecular duplex regions. These initial destabilization events are followed by the rapid sequestration of the unwound sequences into energetically favorable intramolecular structures. For example, the D1-DIS/D2-DIS heteroduplex, upon unwinding, could transition to individual D1 and D2 DIS intramolecular hairpins. Within this framework, the observed resistance of group 2 to DDX3X-mediated unwinding could arise from the thermodynamic barrier associated with reaching the transition state of one of the steps. Indeed, when reconstructing such a multistep process we found that it is possible to unzip group 1 dimer in seven individual steps involving destabilizing short duplexes. Unwinding sufficient base pairs to allow intramolecular structure formation represents ΔG° between −4 and −31.5 kcal (Supplementary Fig. S6). In contrast for group 2, beside the sequences involved in the DIS hairpin and its basal extension, we were unable to identify short duplexes involved in group 2 dimerization and which once unfolded would get trapped into a local intramolecular structure. We make the hypothesis that *in vivo* destabilization of large RNA is possible because of a higher local concentration of DDX3X (we are here under multiple turn-over conditions) or owing to the presence of other single strand binding protein that sequestrates the single strand regions released. Note that this mechanistic model is compatible with generally accepted models and does not imply enzymatic processivity, as individual steps could happen in any order. Up to this point our model suggest that our observations reflect inherent characteristic of the dimer structure rather than DDX3X specificity. However, even considering such a model, the activity observed here remains much higher than that reported by previous studies involving short model duplexes and/or the truncated form of DDX3X [55, 74, 75]. Indeed, the low unwinding activity observed in previous studies led to the constant usage of a large excess of protein to evidence the unwinding activity. This could find its explanation in two specificities of our study. First, in the continuity of the above described model, HIV-1 gRNA appears as a specific substrate, and we previously showed that DDX3X has a high affinity for this substrate (Kd = 30 nM see [46]). Interestingly, high affinity and efficient enzymatic activity are only observed in the presence of the terminal IDRs which is the other specificity of our study. DDX3X IDRs are known to be essential with DDX3X cellular localization and functions as well as with its interaction with specific substrates [7]. In addition to providing specific DDX3X-RNA interaction, we propose that IDR–IDR interactions would allow several DDX3X molecules to work cooperatively on the same RNA molecules even in excess of RNA. Oligomerization through IDR has been postulated in several systems [76, 77] including DDX3X [78]. Moreover, while this manuscript was under consideration, Dharavath *et al.* [79] confirm that DDX3X C-terminal IDR influences DDX3X cooperativity and biochemical activities in conditions of protein excess and in the presence of small duplexes.

This report is also the first example whereby a kinetic approach is used to evaluate the structural rearrangements induced by an RNA helicase. Interestingly, the fast reacting agent BzCN was valuable for both footprinting and kinetic assays, i.e. interactions with differential kinetics. An integrative model of DDX3X interaction with RNA_1–416_ includes the binding of the PBS, DIS destabilization, AUG:U5 dissociation and monomer stabilization. The only apparent discrepancy arises from the lack of reactivity change in the DIS stem during the time-resolved probing experiments. A possible interpretation is that upon DDX3X_WT_ destabilization, SL1 (DIS) elements within intermolecular dimers quickly refold into intramolecular SL1 elements, a phenomenon that could only be trapped by DDX3X_DQAD_ footprinting experiments. In agreement with previous toe-printing experiments [11], SHAPE footprinting identifies the PBS as a specific site. Its important role is further supported by the modeling of the different RNAs used in this study which clearly identifies the PBS stem as one factor that can discriminate between the two RNA populations (Fig. 6). These results are in line with several studies pointing towards the association between the PBS structure and the oligomeric status of HIV-1 gRNA during the viral cycle [34, 80]. Altogether, these results obtained with a biological RNA substrate and the full-length protein challenge our conception of the endowment of DDX3X and pave the way to the reevaluation of DEAD-box proteins activities.

### HIV-1 gRNA structure model

As mentioned previously, HIV-1 gRNA remains a functionally and structurally complex RNA, and the multiple conformations it may adopt are still under debate. In this context, to better appreciate the RNA determinants underlying DDX3X optimal activity, we resorted to an extensive probing of the structures adopted by the different 3′-truncated fragments of HIV-1 gRNA. From this analysis, it can be concluded that HIV-1 dimers can fold into two main conformations that differ by the nucleotides underlying intermolecular base-pairings. The first conformation (**ED1**) corresponds to previously described models whereby intermolecular interactions involve nt 1–343 and is exemplified by the U5:AUG base-pairing. This conformation applies from RNA_1–343_ to RNA_1–942_. The second conformation (**ED2**) predicts that the intermolecular interactions involve nt 1–446. This conformation applies to RNA_1–993_ up to RNA_1–1636_ and indirectly relies on nt 837–868 sequestration by nt 966–981. As this model arises from *in vitro* analyzes, we checked its compatibility with phylogenetic data. A total of 88 sequences representative of each HIV-1 subgroups and SIV.cpz (HIV database Los Alamos) were aligned and the possibility to group 1 and group 2 dimer to form was analyzed. Group 1 could essentially be formed with all sequences but subgroup O’s and group 2 structure was compatible with all subgroups but N, O, and P for which some regions do not base pair (Supplementary Files S4 and S5) and is reminiscent of the conformational changes observed *in vitro* and *in vivo* during the NC-dependent maturation of HIV-1 gRNA dimers [42, 81]. It is thus tempting to speculate that HIV-1 gRNA KL dimers are under the ED1 conformation during packaging and are subsequently remodeled during viral particle maturation to attain the less-reversible ED2 conformation. Recent studies highlight the importance of the TSS on the monomer structure and its propensity to dimerize granting the efficient packaging of the viral genome [32, 34, 82]. Two major populations can be distinguished, the ^Cap^1G population which dimerizes efficiently and is preferentially packaged into virions and the ^Cap^2G/^Cap^3G population whose monomeric conformation restrains dimerization and packaging but has been proposed to be more efficiently translated due to its preferential monomeric conformation (Fig. 1A, monomer [83]). In this study, we used the 2G RNA that has been shown to behave like ^Cap^1G RNA [32] which is essentially under a dimeric conformation therefore rendering the modelization of its monomeric form difficult. Nonetheless, numerous studies performed by the Summers’ lab allowed the proposition of the switch model (Fig. 1A) whereby DIS availability to intermolecular interactions regulates the monomer (U5:DIS)-to-dimer (U5:AUG concomitant to DIS:DIS) transition. Here, we followed 2G RNA dimers destabilization by DDX3X in conditions where dimers cannot re-associate and our results indicate that RNA_1–416_ could go through a transient monomer conformation suggestive of ^Cap^2G/^Cap^3G monomers before attaining the switch monomer exemplified by the U5:DIS interaction.

### Functional consequences

DDX3X has been involved in several aspects of HIV-1 life cycle; although they cannot be conclusive, the *in vitro* results presented here, suggest some interesting avenues to investigate. First, efficient dimer destabilization to the benefit of monomers would favor viral translation [83]. In addition, our results raise the interesting possibility that DDX3X activity could intervene during two crucial steps necessitating remodeling of the PBS structure, i.e. tRNA^Lys, 3^ annealing during viral packaging and initiation of reverse transcription [34, 80, 84]. Altogether our results show that DDX3X displays a specific helicase activity towards HIV-1 dimers and can achieve multiple turn-overs. This specific helicase activity relies on RNA determinants present within the 5′-UTR. More importantly, these results challenge the admitted idea that DEAD-box helicases are rather promiscuous and pave the way for a better understanding of the RNA–DDX3X interface that is crucial to rational drug design.

## Supplementary Material

gkaf834_Supplemental_Files

## Data Availability

The code for nucleotide clustering and scripts used in the analyses are available at https://github.com/gdebissc/DDX3. All other data underlying this article are available in the article and in its online supplementary material.
