# Supplementary material for: DEAD-box helicase intrinsically disordered domains and structural dynamics of HIV-1 RNA are required to reveal DDX3X catalytic efficiency
Source: Nucleic Acids Res. 2025 Aug 28;53(16):gkaf834. doi: 10.1093/nar/gkaf834 (PMC12392093; doi:10.1093/nar/gkaf834)
Supplement: gkaf834_Supplemental_Files [file gkaf834_supplemental_files.zip › supplementary_material.pdf]

Supplementary materials for

DEAD-box Helicase Intrinsically Disordered Domains and Structural Dynamics of HIV-1 RNA are Required to reveal DDX3X Catalytic Efficiency

Chamond *et al*.

Corresponding authors Email: nathalie.chamond@u-paris.fr, bruno.sargueil@u-paris.fr

This pdf includes

Oligonucleotides used in this study

Figs S1 to S6

Tables S1 to S4

Oligonucleotides used in this study

T7_F (5’-TAATACGACTCACTATAGGTCTCTCTGGTTAGACCAGATCT-3’), 223_R (5’-CTCTGGCTTTACTTTCGCTTTCA-3’), 343_R (5’-GCACCCATCTCTCTCCTTCTAGC-3’), 416_R (5’-TTTCTTTCCCCCTGGCCTT-3’), 540_R (5’-GAAGGGATGGTTGTAGCTGTCC-3’), 851-R (5’-AAACATGGGTATTACTTCTGG-3’), 875_R (5’-GGTGGCTCCTTCTGATAATGC-3’), 942_R (5’-CTTTTAACATTTGCATGGCTGC-3’), 993_R (5’-CATGCACTGGATGCAATCTATCC-3’), 1040_R (5’-GTCACTTCCCCTTGGTTCTCTC-3’), 1074_R (5’-TTTGTTCCTGAAGGGTACTAGTAG-3’), 1396_R (5’-CCCACTCCCTGACATGC-3’), 1636_R (5’-AAAAAATTAGCCTGTCTCTCAGTACAATC-3’).

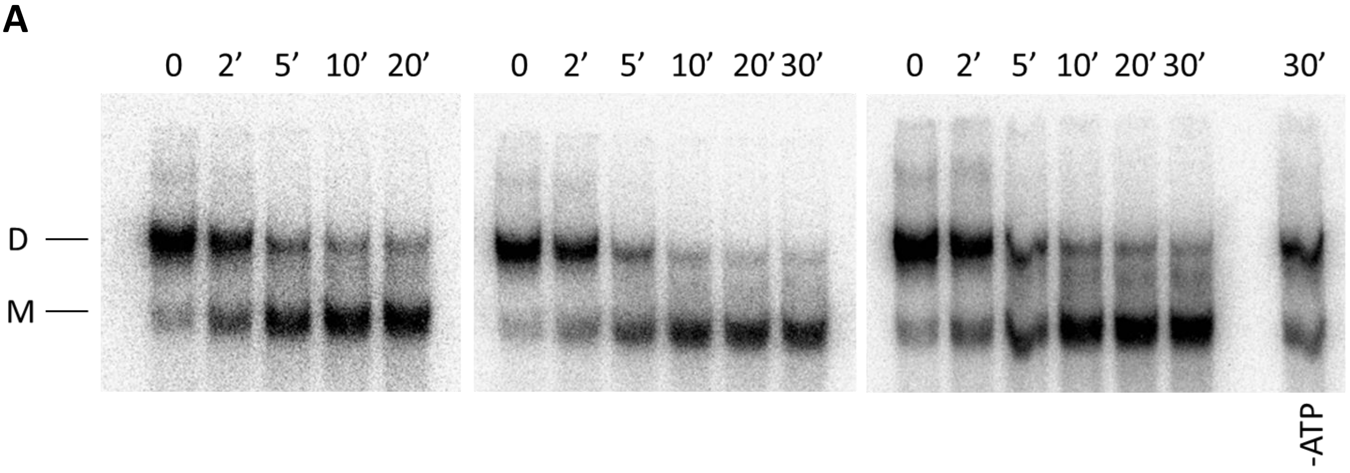

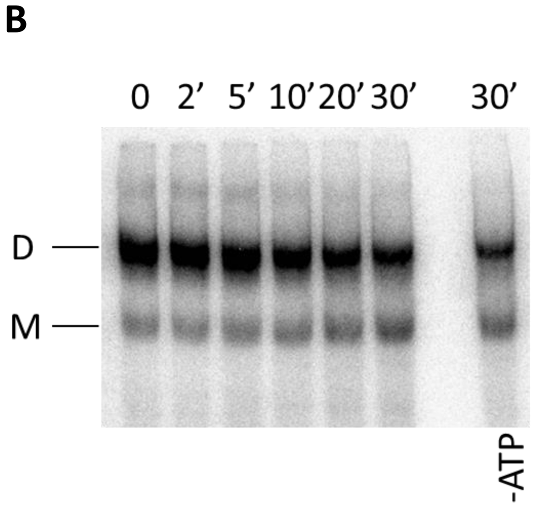

Figure S1. Native gel electrophoresis of the time-course of $RNA_{1\text{-}416}$ (50 nM) dimer destabilization by (**A**) $DDX3X_{WT}$ (20 nM) or (**B**) Dbp7 (20 nM) in the absence or in the presence of 2 mM ATP.

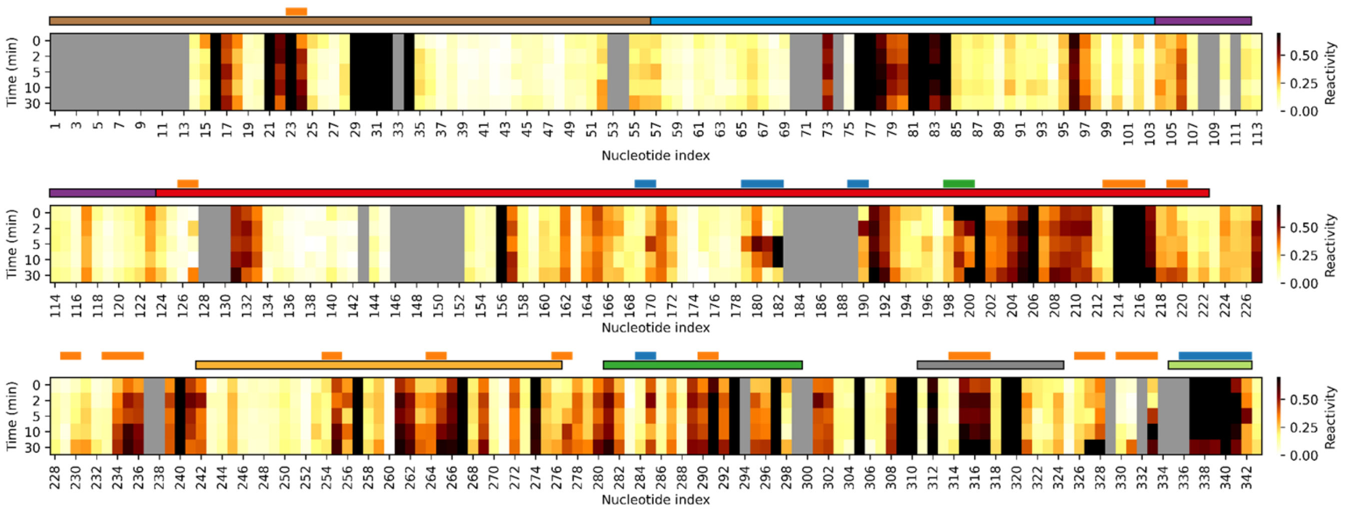

Figure S2. BzCN reactivities of HIV-1 5'UTR (50 nM) in the presence of DDX3X (5 nM) at different timepoints. Grey indicate undetermined reactivities. Annotations on top of the heatmaps indicate the secondary structure elements, colored as in Figure 1, and nucleotide clusters colored as in Figure 3 (blue: cluster 0, orange: cluster 1, green: cluster 2)

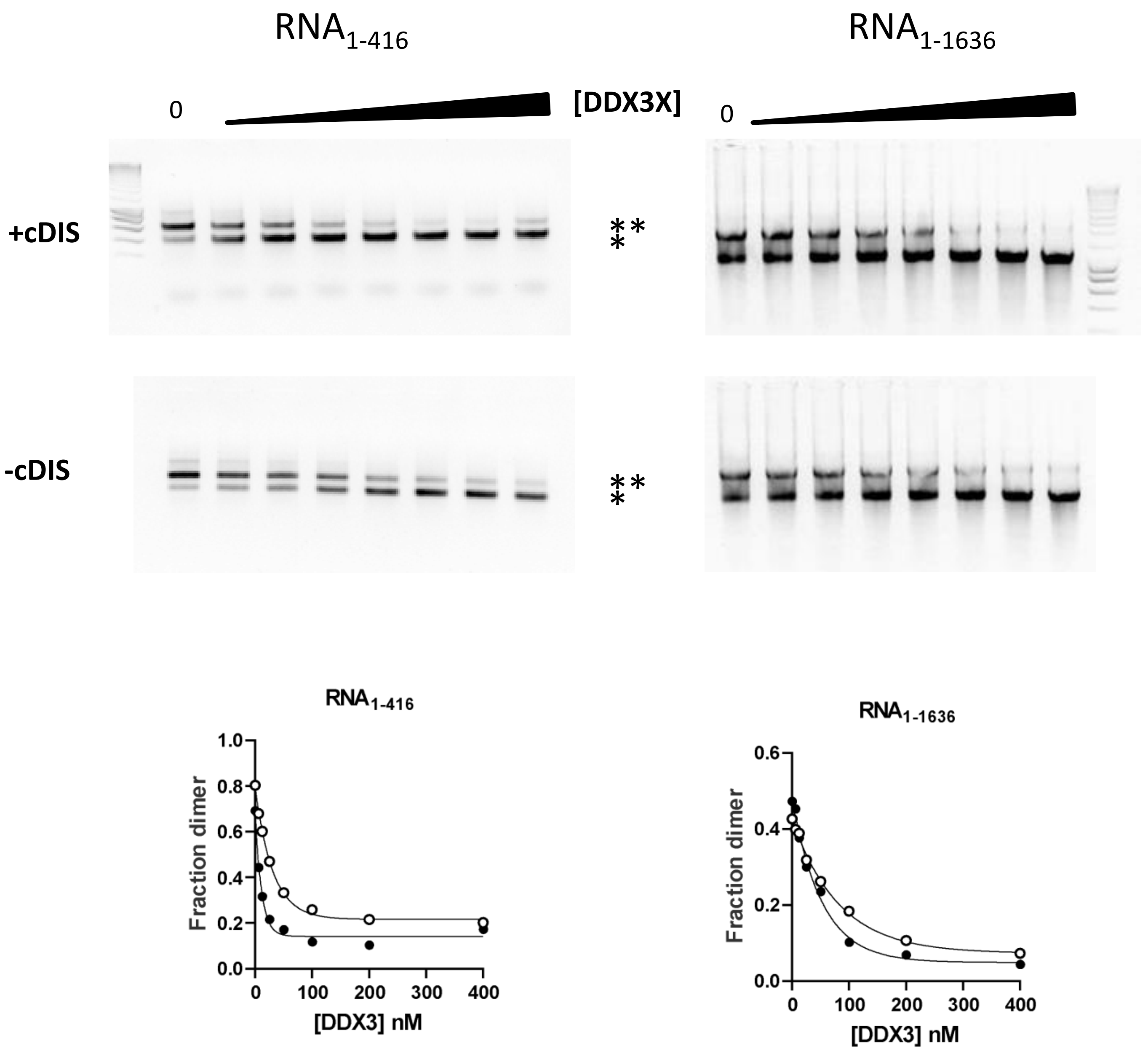

Figure S3. DDX3X dimer destabilisation in the presence (closed circles) or absence (open circles) of 2 µM anti-DIS oligonucleotide. 0-400 nM DDX3X WT were incubated in the presence of 400 nM RNA for 10 min at 37°C in Helicase Buffer. Reactions were stopped and loaded onto 1 % agarose gel and stained with EtBr. **, dimers; * monomers.

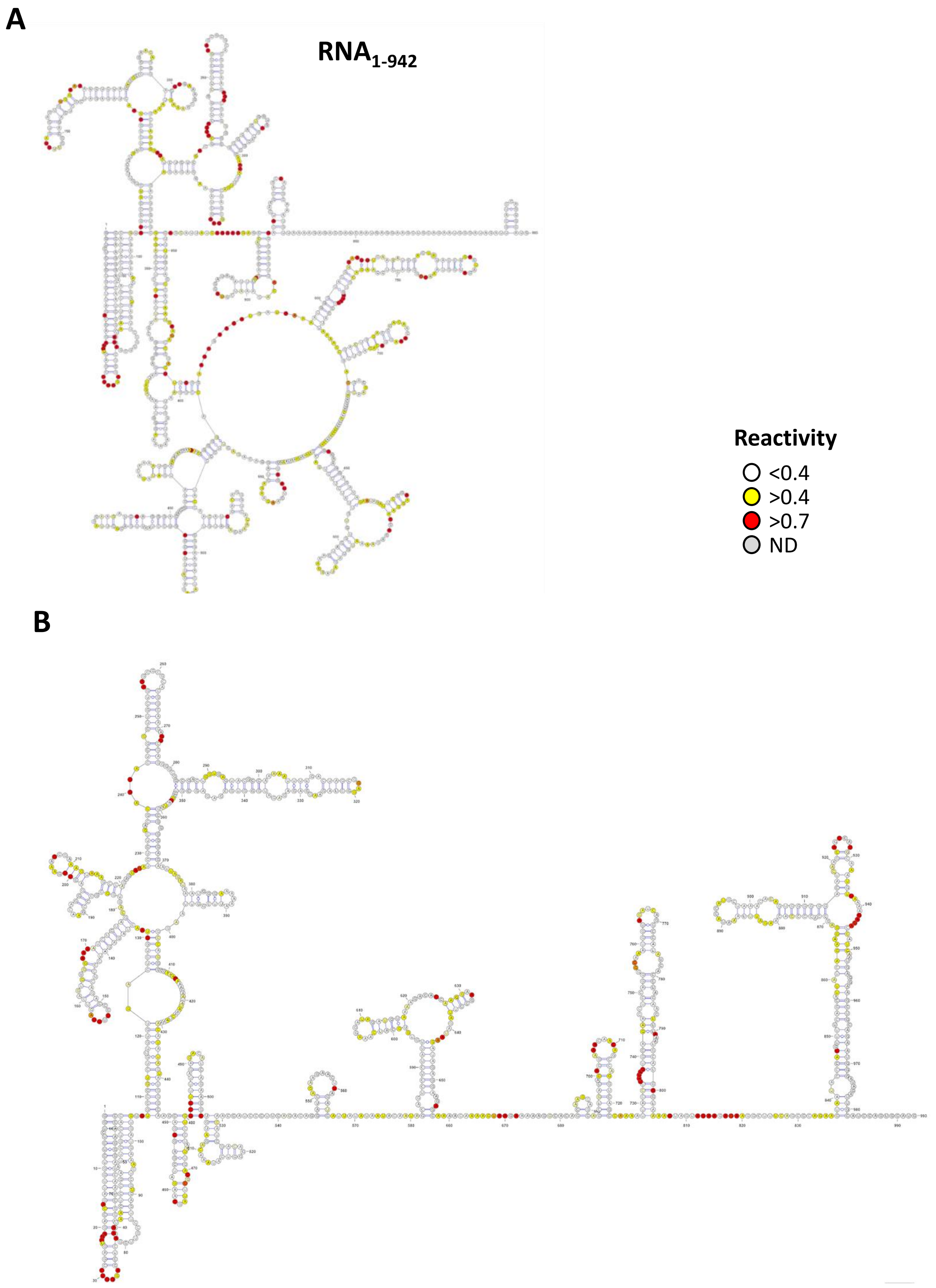

Figure S4. The secondary structure of HIV-1 5’UTR was modeled with IPANEMAP using the measured reactivities as indicated in the legend (mean and s.e.m. indicated in Table S1). Dimeric RNAs are represented as monomers for clarity sake.

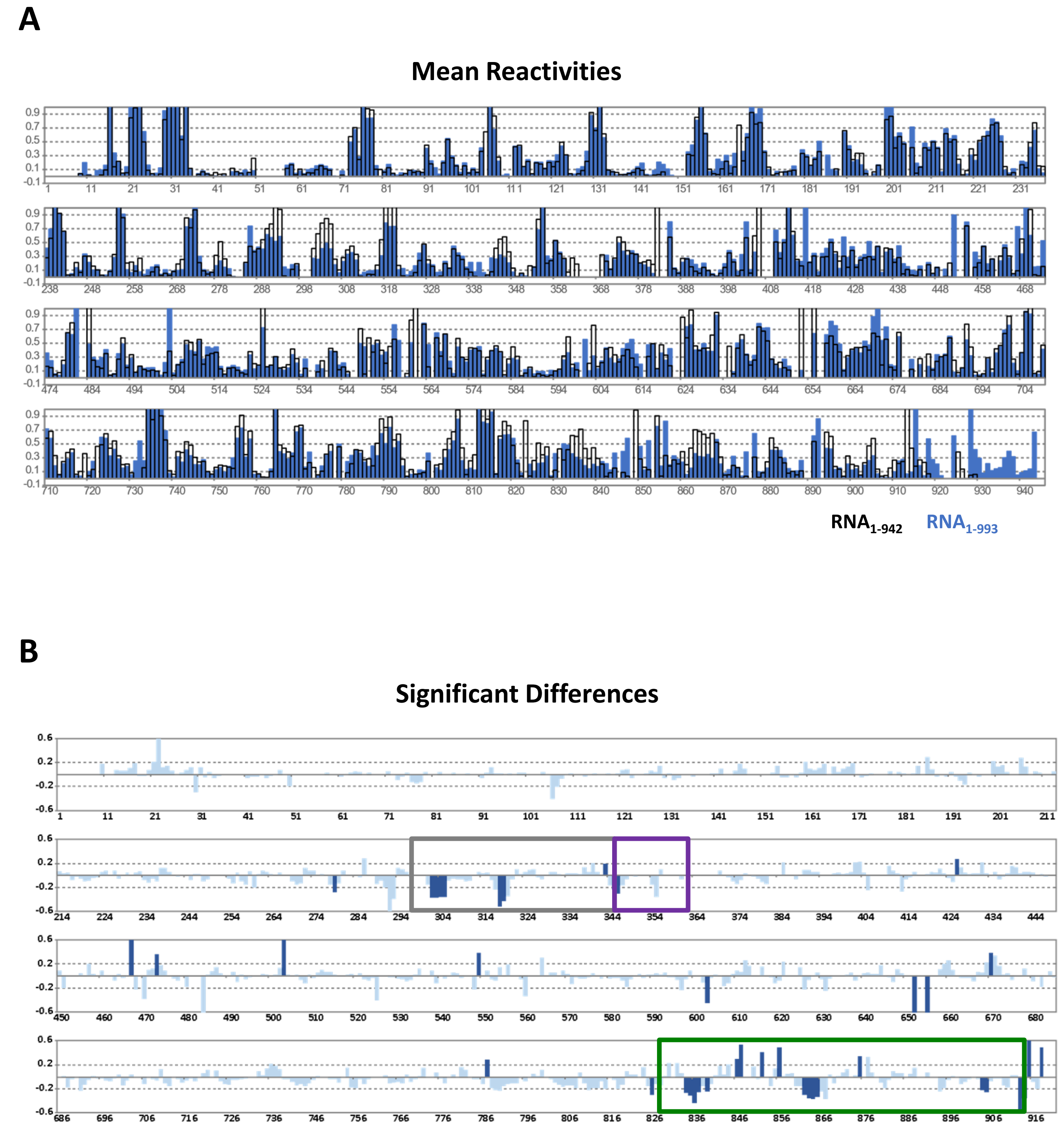

Figure S5. (**A**) Surimposition of the reactivity profiles obtained for $RNA_{1\text{-}942}$ and $RNA_{1\text{-}993}$ and (**B**) analyzed as described in (1).

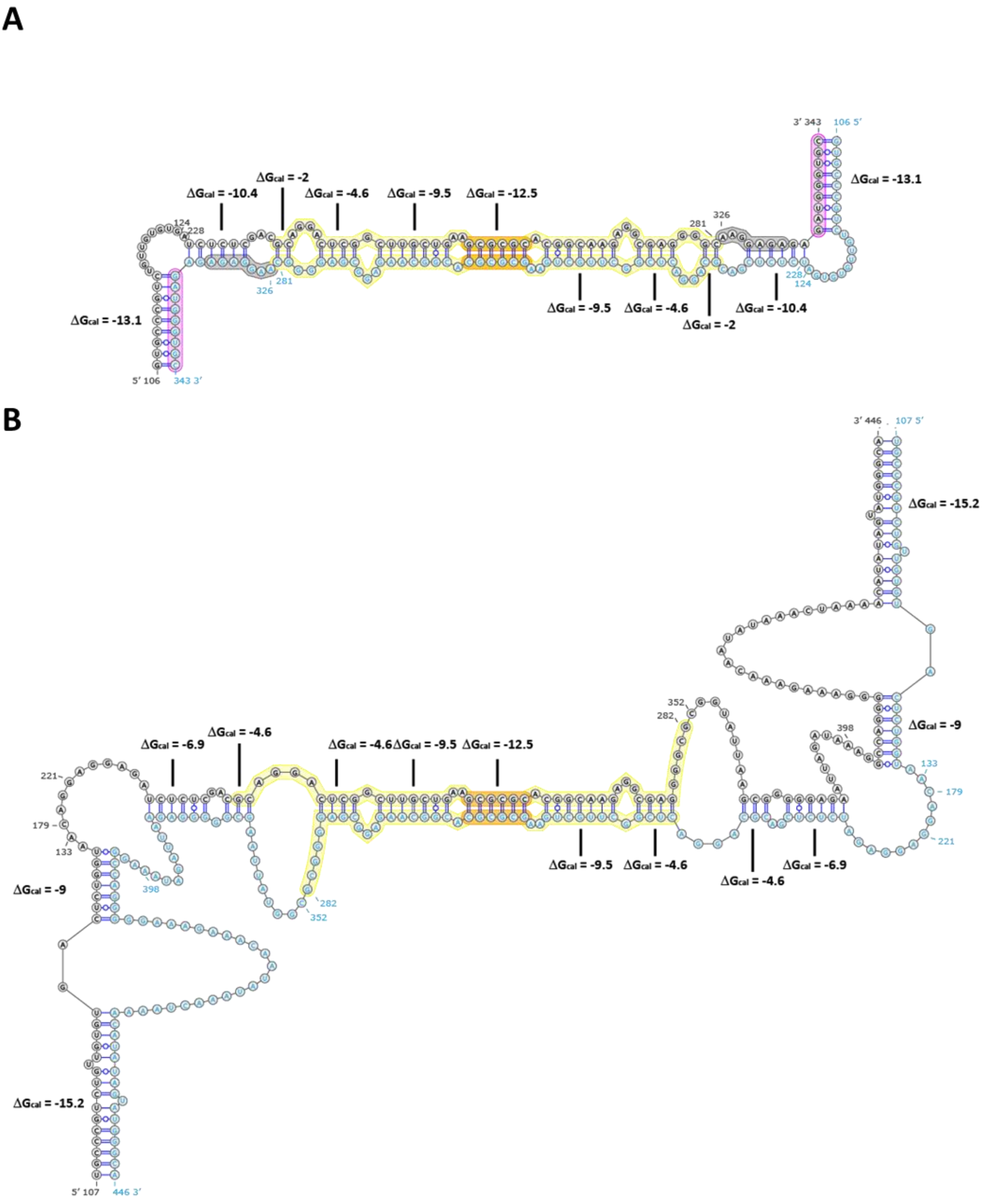

Figure S6: Intermolecular interactions in **A**) ED1 ($RNA_{1\text{-}942}$) or **B**) ED2 ($RNA_{1\text{-}993}$) dimers and calculated helices stability (DG_{calc} $kcal.mol^{-1}$). Colored nucleotides correspond to region that can fold locally (DIS, yellow ; Kissing Loop, orange ; Psi, grey ; AUG/SL4, pink). Nucleotides involved in intramolecular interactions are not represented for clarity sake.

References

1. de Bisschop,G. and Sargueil,B. (2021) RNA Footprinting Using Small Chemical Reagents. Methods Mol Biol, 2323, 13–23.

Table S1. (separate file)
Probing performed in this study

Table S2. (separate file)
Helicase assays performed in this study

RNA	Km (nM)<br>Mean ± s.e.m.	Vmax (nM.min^{-1})<br>Mean ± s.e.m.	N
RNA_{1-343}	40.3 ± 3.8	4.9 ± 0.4	6
RNA_{1-416}	45.5 ± 6.3	4.7 ± 0.4	5
RNA_{1-540}	55.1 ± 10.7	4.2 ± 0.7	3
RNA_{1-851}	37.8 ± 4.7	4.2 ± 0.2	4
RNA_{1-1074}	29.3 ± 5.6	2.0 ± 0.3	4
RNA_{1-1396}	39.5 ± 8.7	1.3 ± 0.7	3
RNA_{1-1636}	48.9 ± 5.1	1.3 ±0.3	3

Table S3: DDX3X unwinding activity towards HIV-1-derived dimers. The results are the mean and standard deviation of three to six independent experiments.

RNA	Km (nM) Mean ± s.e.m.	Vmax (nM.min^{-1}) Mean ± s.e.m.	N
RNA_{1-875}	67.6 ± 12.0	5.4 ±0.5	3
RNA_{1-942}	70.5 ± 7.8	5.8 ± 1.1	3
RNA_{1-993}	22.0 ± 5.7	1.8 ± 0.1	3
RNA_{1-1040}	39.4 ± 12.4	2.1 ± 0.5	4

Table S4: DDX3X unwinding activity towards HIV-1-derived dimers. The results are the mean and standard deviation of three to four independent experiments.

Supplementary files S5 and S6 (separate files)

Group 1 (S5) and group 2 (S6) secondary structure alignment of a selection of representative HIV-1 and SIV.Cpz. Available representative sequences for the 5'UTR and Gag sequences for each HIV-1 subgroup and SIV.cpz were recovered from the Los alamos HIV sequence database (RIP alignment - https://www.hiv.lanl.gov/content/sequence/NEWALIGN/align.html). They were then trimmed to retain only bases involved in group 1 or group 2 dimer formation. They were then aligned in regard to base pairing of our models encoded with dot and brackets. Files provided are the corresponding stockholm files.
